# Health-Related Factors Influencing Nurse Turnover by Clinical Career: A Secondary Data Analysis of Clinical Nurses in South Korea

**DOI:** 10.3390/ijerph192215222

**Published:** 2022-11-18

**Authors:** Jiwon Kang, Youngjin Lee

**Affiliations:** 1 Research Institute of Nursing Science, Ajou University, Suwon 16499, Republic of Korea; 2 School of Nursing, University of Minnesota, Minneapolis, MN 55455, USA; 3 College of Nursing, Ajou University, Suwon 16499, Republic of Korea

**Keywords:** survival analysis, turnover, clinical career, nurse

## Abstract

The increase in clinical nurse turnover is an important issue in human resource management worldwide. Factors influencing it include health-related risk factors such as sleep quality and presenteeism, which need further exploration. We examined differences in job survival time of clinical nurses in relation to nurses’ sleep quality and presenteeism. Participants were 857 Korean clinical nurses with more than three months’ experience providing direct patient care. Data were analyzed using a time-independent Cox proportional hazard regression analysis of factors affecting actual turnover of clinical nurses. Average job survival times of competent, proficient, and expert nurses were 33, 64, and 143 months, respectively. Sleep quality and presentism significantly affected turnover risk. For clinical nurses with less than three years of experience, sleep quality significantly influenced the risk of turnover. For clinical nurses with more than six years of experience, presenteeism significantly affected the risk of turnover. The findings of this study offer a clinical career-based approach to reduce the turnover rate of clinical nurses. A differentiated approach based on work experience is necessary to establish a turnover management strategy for clinical nurses.

## 1. Introduction

As professionals who provide integrated care to patients, clinical nurses have received increased attention due to their essential role in society. With the growing demand for clinical nurses, the Korean government has been gradually allowing nursing departments to increase their entrance quotas. This has led to an approximately two-fold increase in nursing quotas over a relatively short period of time [1]. The current trend of increasing numbers of nursing graduates is expected to lead to an oversupply of nurses by 2030 [2]. In addition, over the past 20 years, the total number of male nurses (cumulative) has increased significantly from 1795 (1.5%) in 2010 to 10,965 (5.1%) in 2020 [3]. However, the short-term increase in the number of licensed nurses has been associated with a steep increase in the annual nurse turnover rate, as newly registered nurses leave the profession, being unable to adjust to the workplace [4,5]. Filling the gap in work due to high turnover rates and repeating the preceptorships of other newly registered nurses ultimately increases the burden on existing experienced nurses. This continuously puts a burden on professional nurses and leads to a vicious cycle that increases their turnover rate [6]. Additionally, nurse turnover negatively affects patient treatment outcomes as it increases inadequate nursing care and leads to an increase in the incidence of accidents, infections, readmissions, and preventable mortalities [7]. Therefore, an increased turnover rate reduces the overall quality of nursing care and negatively affects patient-centered nursing [8].

Clinical nurses who engage in shift work are exposed to diverse health statuses caused by irregular sleep patterns that reduce sleep quality and increase various health risks, including physical and psychological health [9,10]. Health problems for clinical nurses caused by reduced sleep quality prevent the completion of given tasks and have a strong influence on nurse turnover [11]. This has significant implications for the health status and lives of existing experienced nurses engaged in long-term shift work [12] as well as newly registered nurses facing new sleep patterns [13]. These factors influence the continuity of work for clinical nurses, indicating the need to study nurse turnover as it relates to sleep quality, based on career duration.

Presenteeism occurs when a worker is required to work despite a health problem that demands rest from work, resulting in a negative effect on productivity [14]. Clinical nurses show a high level of presenteeism as their tasks involve complex relationships with different public health workers, health issues due to shift work, excess workload, high stress levels, and increased demand for patient care [15]. The outcomes of presenteeism have a serious impact on patient outcomes, leading to a decline in nurse health and well-being [16,17] as well as the turnover problem of clinical nurses [18]. An accurate understanding of the clinical significance and occurrence of presenteeism is essential, as detection and early intervention can prevent progression over time [19].

Although there have been studies on the extent to which changes in sleep quality or presenteeism are related to clinical nurses’ turnover, few studies have examined whether these factors affect nurse turnover according to career duration. This is a major problem ailing many organizations, as identifying the turnover intention according to nurses’ career duration can provide insights for human resource management activities and prevent higher costs and losses [10]. Therefore, this study evaluated the survival time of nursing career and analyzed the differences in survival time according to sleep quality and presenteeism to identify the factors affecting nurse turnover. This approach can help identify health and turnover-related factors for the continuity of work for clinical nurses, prevent turnover, and promote healthy lifestyles throughout the shift nurse’s career.

## 2. Methods

### 2.1. Design

This study involved a cross-sectional secondary analysis of longitudinal data from a nurse compassion competence study conducted in South Korea [20,21]. Briefly, the parent study aimed to investigate the effects of compassion competence on the quality of care and the nursing profession and establish a compassion competence prediction model among clinical nurses. Korean clinical nurses’ turnover status and turnover survival were analyzed using annual turnover survey data from this three-year longitudinal study.

### 2.2. Participants

The nurse compassion competence study enrolled 2041 clinical nurses [21]. The inclusion criteria for participants of the study were clinical nurses who (a) worked in an acute general or long-term care hospital that could accommodate more than 100 beds in any city in Korea, (b) provided treatment directly to patients, and (c) had spent more than three months of their career providing patient care. In this study, the following inclusion criteria were used to select participants for analysis from the parent study data [21]: (1) nurses with as many as three years of follow-up monitoring; and (2) those with an apparent answer to all relevant items (total clinical career, health-related characteristics, status of turnover, and date of turnover). Although 1556 participants were considered for the analysis, among them, only data from 857 participants, excluding dropouts during the entire study period, were used for the final analysis during the total study period. We named the careers based on Benner’s philosophy in nursing practice [22]. Participants in this study were clinical nurses with at least 3 months of experience, and among Benner’s five stages, the novice (all students or any nurse entering a clinical setting with no clinical experience with that particular patient population) and advanced beginner (the nurse will focus on the rules and have developed an initial understanding of patterns of care) were judged to have already been sufficiently passed; the subjects were divided into three stages: competence, proficiency, and expertise. Therefore, we named those with (1) less than three years of experience: competent nurses; (2) 3–6 years: proficient nurses; and (3) six years or more: expert nurses.

### 2.3. Data Collection

Participant data were derived from answers to the self-administered questionnaire that was conducted over three years, using the online data collection tool SurveyMonkey, from February 2018 to February 2020. This included the Korean version of the Pittsburgh Sleep Quality Index (PSQI-K) [23,24], Stanford Presenteeism Scale (SPS) [25], demographic data, health status, total clinical career length, status of turnover, and date of turnover. The data used in this study were based on answers to all relevant topics in the questionnaire, which were tracked for up to three years. It was announced in advance that a three-year follow-up investigation will be conducted when collecting data for the first year to track the subjects, and annual discrimination data were collected by checking attendance at SurveyMonkey. Therefore, the 3-year data could be collected consistently with the initial dataset, and quality was guaranteed.

### 2.4. Ethical Considerations

This study was conducted after obtaining approval from the institutional review board (AJIRB_MED_MDB_19_392). To protect the rights of the participants, a joint investigator, unrelated to the participants, provided information about the study and provided informed consent for the use of secondary data. Personal information was deleted from the dataset used for the analysis.

### 2.5. Data Analysis

Data were analyzed using R version 4.0.2. For general participant characteristics, descriptive statistics were used to calculate the frequency, percentage, mean, and standard deviation. Additionally, *t*-tests and χ^2^-tests were performed to examine the differences in characteristics between the turnover and non-turnover groups.

Data for the dependent variable (actual turnover) and independent variables (sleep quality, current) were extracted from the entire dataset and loaded in Python DataFrame format. Exploratory data analysis was performed using Python 3.7, NumPy, Pandas, and Matplotlib. Moreover, a test of model suitability was conducted using the R 4.0.2 package, survival, survminer, dplyr, and glmnet. For PSQI and presenteeism as analysis targets, the model suitability and significance of the regression coefficient were tested. Finally, the selected model was tested using a time-independent Cox proportional hazard regression model. A proportional hazard hypothesis test was conducted using this model. When no hazard was found, the influence of the specific subitems for each covariant was determined using stepwise variable selection based on partial likelihood.

### 2.6. Instruments

#### 2.6.1. Sleep Quality

Sleep quality and sleep patterns were evaluated using the PSQI-K translated and validated by Sohn et al. [23] based on the original PSQI by Buysse et al. [24]. This instrument consisted of 18 self-reported items related with sleep quality, sleep onset latency, sleep duration, sleep efficiency, sleep disturbance, use of sleeping medication, and daytime dysfunction. Each item was rated on a four-point scale from 0 (never) to 3 (more than three times a week). The PSQI-K global score ranges from 0 to 21, and this result comes from seven components about sleep and daytime function, with higher scores indicating worse sleep quality. Based on the cut-off point by Buysse et al. [24], we classified participants with PSQI scores of ≤5 as having good sleep quality and those with a score of >5 as having poor sleep quality. Cronbach’s alpha coefficient values were 0.84 for the original PSQI-K [23] and 0.76, respectively.

#### 2.6.2. Presenteeism

Turpin et al. [25] designed the SPS to estimate work loss attributed to absenteeism and presenteeism, precisely due to one of several potential health conditions. Participants were guided to select health-related problems that they were concerned about from a list of issues related to work loss. Next, the participants were asked about the influence level of the selected problems on their work, measured using a 5-point scale for a total of 10 questions. Each question was evaluated from a score of 1 (not at all) to 5 (at all times). Higher scores indicated higher levels of work loss.

According to the scale developer’s recommended methods, work loss in this study was calculated using the following 100-point conversion equation: (total score − 10)/40 × 100 points.

#### 2.6.3. Sociodemographic Characteristics

The participants’ general characteristics included gender, age, marital status, religion, education, and self-rated health status. Furthermore, we analyzed work-related characteristics such as work departments, clinical careers, turnover plans, and actual turnover.

Concerning the time variable, although the original data reported clinical career in years and months (e.g., three years and two months), these values were converted to months for this analysis. The total clinical career in months was used as the time variable when censoring cases were defined in the third year of the project. The nurse’s entire clinical career in months in the second year was used as the time variable in cases where the response to the turnover plan item was “yes.” The date of resignation was recorded in the second year, or censoring was defined in the second year; no response was found in the third year.

Survival time in this study refers to the time during turnover when the participant responded to confirm the status of turnover or resignation. It was indicated by the total clinical career up to the time of the survey, based on a response of “yes” to the turnover plan item, with a recorded date of resignation.

Censoring, as used in this study, refers to cases of non-turnover at the time of the survey, where the status of turnover or resignation for the participant could not be confirmed. It was based on a response of “no” to the turnover item.

The variables for defining censoring relied on a negative response to a single item (“Have you been part of a turnover in your job in the past year?”) to determine turnover. In cases with a negative response, we rechecked and confirmed that no data were recorded under the “resignation date”.

## 3. Results

### 3.1. Demographic Characteristics

The final analysis included data from 857 clinical nurses (Table 1). These clinical nurses were classified by turnover status as follows: 284 (actual turnover group) and 573 (non-turnover group). No significant difference in age was found between the turnover and non-turnover groups (29.1 and 29.0 years, respectively; *p* = 726).

Based on gender, the turnover group included 11 men and 273 women, whereas the non-turnover group included 22 men and 551 women. Therefore, no statistically significant differences were found between the two groups regarding gender (*p* = 0.869). The median survival time for female nurses was 133 months (95% confidence interval [CI]: 133.0, 141.0). For male nurses, the median survival time could not be estimated because the minimum survival was ≥0.5 (probability of survival; 0.5 = 50%; estimated when the survival rate (not yet turnover) of all participants reaches 50%). No statistically significant gender differences were found between male and female nurses (*p* = 0.487).

Regarding marital status, a statistically significant difference was found between the turnover and non-turnover groups (*p* = 0.04). The median survival time for single nurses was 123 months (95% CI: 109.0, 133.0). The median survival time could not be estimated for married nurses as the minimum survival was ≥0.5% (probability of survival; 0.5 = 50%). Single and married nurses showed statistically significant differences. The median survival time for single nurses was found to be lower than that for married nurses.

With regard to nurses’ educational background, no difference was found between the turnover and non-turnover groups regarding those with a degree of three years or less and a degree of four years or more (*p* = 0.507). The estimated median survival time for clinical nurses with a degree of three years or less was 141 months (95% CI: 133.0, 143.0), whereas that for those with a degree of four years or more was 133 months (95% CI: 121.0, 134.0). A statistically significant difference was found between clinical nurses with a degree of three years and those with a degree of four years (*p* = 0.02). The more educated group showed a shorter median survival time than the less-educated group.

When studying actual turnover, the ratio of clinical nurses with turnover plans and clinical nurses without turnover plans showed a statistically significant difference. The median survival time could not be estimated for clinical nurses without turnover plans, as the minimum survival was ≥0.5 (probability of survival; 0.5 = 50%). However, for clinical nurses with turnover plans, the estimated median survival time was 121 months (95% CI: 104.0, 133.0). Clinical nurses with turnover plans and those without turnover plans showed a statistically significant difference. Overall, the median survival time was longer for nurses with turnover plans than for those without plans.

The health status of clinical nurses was also examined. The ratio of clinical nurses with good health (the group with a high health status score in the survey) and clinical nurses with poor health (the group with a low health status score in the survey) showed a statistically significant difference between the turnover and non-turnover groups. The median survival time was 142 months (95% CI: 134.0–143.0) for clinical nurses with good health. However, for clinical nurses with poor health, the median survival time was 130 months (95% CI: 115.0, 133.0). Therefore, with regard to health status, a statistically significant difference was found between clinical nurses with good health and those with poor health. Overall, clinical nurses with poor health had shorter median survival times than those with good health.

For clinical career, a statistically significant difference was found between the turnover and non-turnover groups when stratified by years spent in the career of nursing (less than three years = competent nurses; three to six years = proficient nurses; and six years or more = expert nurses). The median survival time was 33 months, 64 months, and 143 months for competent (95% CI: 29.0, 34.0), proficient (95% CI: 61.0, 67.0), and expert nurses (95% CI: 134.0, 143.0), respectively. There was a statistically significant difference between clinical nurses with different career lengths.

### 3.2. Job Survival Time (Actual Turnover)

**Figure 1:**
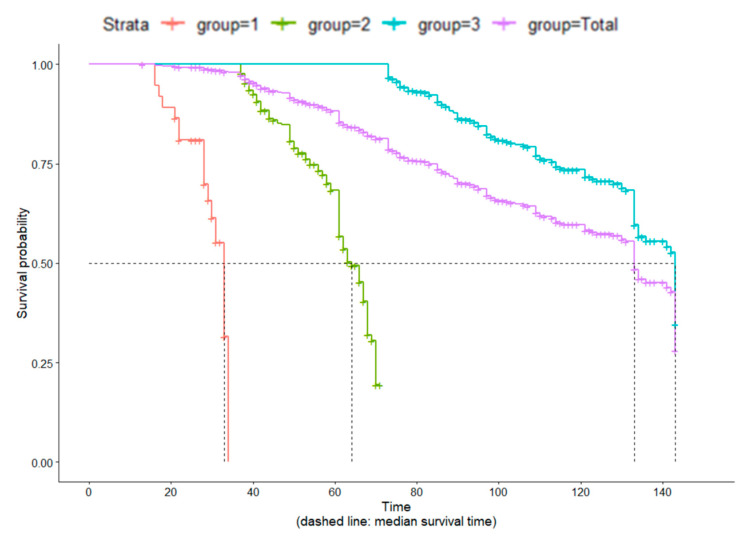
Survival curves based on career duration of nurses. The purple line denotes all nurses (group = total). The red line denotes competent nurses (group = 1). The green line denotes proficient nurses (group = 2). The blue line denotes expert nurses (group = 3).

Survival curves were constructed based on nursing career duration. This allowed for the analysis of the survival time of actual turnover (Figure 1). According to the survival curve for the career duration of all clinical nurses (group = total), the estimated median survival time was 133 months (95% CI: 133, 143). The median survival time was 33 months, 64 months, and 143 months for competent (95% CI: 29.0, 34.0), proficient (95% CI: 61.0, 67.0), and expert nurses (95% CI: 134.0, 143.0), respectively.

### 3.3. Factors Influencing Turnover: Time-Independent Cox Proportional Hazard Regression

For the cohort of all clinical careers, the result of selecting the variables based on partial log-likelihood showed that the time-independent Cox proportional hazard regression with the two variables of sleep quality and presenteeism was statistically significant, as verified by the result of a goodness-of-fit test (see Table 2). In this model, sleep quality significantly affected the risk of turnover (HR, 1.28). Notably, the more negative the response was to the fifth subitem of presenteeism (“I had difficulty controlling work stress as I was concerned with a health issue”), the higher the relative risk of turnover (HR, 1.13).

For the cohort with a clinical career of three years or less, the result of selecting the variables based on partial log-likelihood showed that the Cox proportional hazard regression with the variable of sleep quality was statistically significant, as verified by the result of a goodness-of-fit test. In this model, sleep quality significantly affected the risk of turnover (HR, 9.16). Notably, the more negative the response was to the first and third subitems of sleep quality (subjective sleep quality: HR, 2.92; and sleep time: HR, 2.17), the higher the relative risk of turnover. For the cohort of proficient nurses, the risk of turnover could not be adequately estimated using the main variables. For the cohort of expert nurses, the result of selecting the variables based on partial log-likelihood showed that the Cox proportional hazard regression with two variables, sleep quality and presenteeism, was statistically significant, as verified by the result of a goodness-of-fit test. Presenteeism (HR, 1.31) substantially affected the risk of turnover. Notably, the more negative the response was to the fifth subitem of presenteeism (“I had difficulty controlling work stress as I was concerned with a health issue”), the higher the relative risk of turnover (HR, 1.22).

### 3.4. Survival Curve and Cumulative Probability of Turnover by Presenteeism and PSQI Score

**Figure 2:**
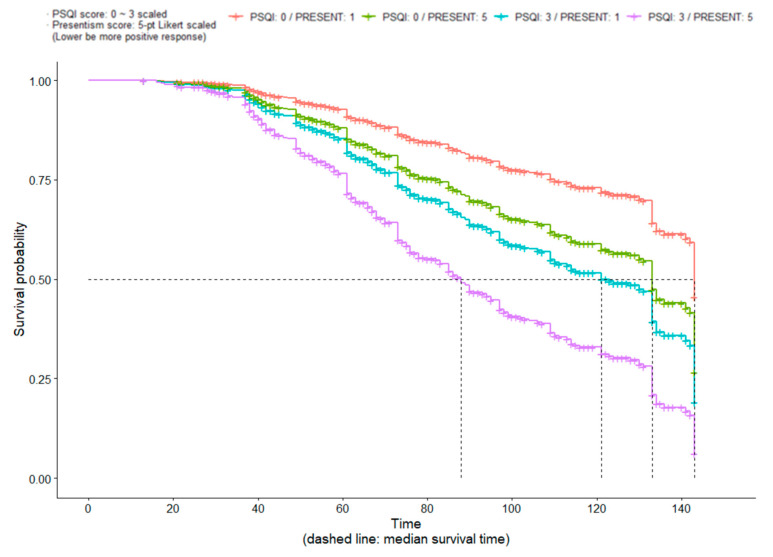
Survival curves based on career duration of nurses according to PSQI and presenteeism. A PSQI score of 0 was the minimum value considered (red, green), whereas 3 was the maximum (purple, blue). A presenteeism (PRESENT) score of 1 was the minimum (red, blue), whereas 5 was the maximum (green, purple).

Survival curves were constructed to determine the median survival time based on presenteeism and the PSQI model for all clinical nurses (Figure 2). Using the Cox proportional hazard model validated for all study participants, the Kaplan–Meier results of predicted conditional probability of survival were visualized in the following four groups: (1) PSQI_low_ + Presenteeism_low_ group; (2) PSQI_low_ + Presenteeism_high_ group; (3) PSQI_high_ + Presenteeism_low_ group; and (4) PSQI_high_ + Presenteeism_high_ group. Thus, a visual presentation was obtained for the regression model of turnover risk with two explanatory variables: PSQI and presenteeism. The estimated median survival time was the longest at 143 months (95% CI: 141, ∞) for the PSQI_low_ + Presenteeism_low_ group, followed by 133 months (95% CI: 103, ∞) for the PSQI_low_ + Presenteeism_high_ group and 121 months (95% CI: 89, ∞) for the PSQI_high_ + Presenteeism_low_ group. It was the shortest at 88 months (95% CI: 73, ∞) for the PSQI_high_ + Presenteeism_high_ group.

## 4. Discussion

In this study, we investigated factors affecting turnover among nurses by evaluating the survival time of nursing career and analyzing the difference in survival time according to sleep quality and presenteeism.

Clinical nurses with turnover plans had shorter survival times than those who did not. In particular, newly licensed registered nurses showed a higher level of actual turnover because they experienced sleep problems during their early career period [26]. This led them to set up a turnover plan before adjusting to a new nursing environment and achieving job satisfaction [27]. The duration of field training for newly registered nurses in Korea is typically less than two months [6], and it is during this time that new nurses form a turnover plan [6]. A novel approach is needed to help newly assigned clinical nurses. Using specific programs designed to help clinical nurses adapt to the hospital environment could perhaps be effective [28].

Clinical nurses with poor health had significantly shorter retention times than those with good health. Most clinical nurses performing shift work reported experiencing various health statuses simultaneously. Moreover, sleep disturbance, fatigue, and depression are essential factors in the turnover [29]. As the maintenance of good health is directly related to turnover, the health status of clinical nurses should be periodically and systematically reviewed as part of human resource management in hospitals.

For the cohort of all clinical careers, sleep quality in clinical nurses was found to affect turnover significantly. For clinical nurses who work shifts and are not guaranteed good sleep quality, the lifestyle demanded by their work can deteriorate their health and lead to a higher risk of turnover [26]. In most cases, it is impossible to change the schedule composition of the work team; therefore, nursing team leaders can encourage nurses to use a daily nap system. In addition, individual and family rhythms should be adjusted to suit various work schedules and days of the week, and deviations in sleep clock rhythm should be reduced [30]. Similarly, to ensure that patients receive quality care, nurses can organize night shifts such that they include naps lasting more than an hour for greater recovery after the shift [31]. Therefore, the starting point for managing nurse turnover levels is to positively promote clinical nurses’ health and foster effective job performance, including bolstering strategies to measure and improve clinical nurses’ sleep quality, such as sleep interventions [32].

Notably, for the cohort of competent nurses, sleep quality significantly affected turnover risk. Relative risk was shown to increase in clinical nurses with low subjective sleep quality or short sleep duration. This is in line with the finding among newly registered nurses in the first two years of work, as we found that the turnover rate was higher in the high-symptom group with two distinct sleep disorders than in the low-symptom group. Additionally, sleep disorders were more frequent among clinical nurses with high symptoms in the turnover group than in the non-turnover group [13]. New clinical nurses who have undergone a challenging adaptation period are likely to have more severe sleep-related problems [33]. If such problems persist, clinical nurses may choose to end their careers. Therefore, because clinical nurses experience sleep problems in the early years of their careers, clinical managers should directly engage in problem-solving by providing more efficient scheduling and focusing on helping newly registered nurses adapt to the new environment.

For the cohort of total clinical careers and expert nurses, the relative risk of turnover increased as responses became more negative toward the fifth subitem of presenteeism (“I had difficulty controlling work stress as I was concerned with a health issue”). Anxiety-related physical symptoms and work role stress were reported by individuals who expressed a level of emotional fatigue, and health care workers who were emotionally worn out showed a higher rate of absence and turnover [34,35,36]. Thus, it is necessary to develop a practical measure to help clinical nurses control work-related emotional fatigue and stress. Notably, professional resource managers at hospitals are required to manage factors associated with turnover and work stress that arise in association with the psychological health of clinical nurses to promote work efficiency.

### Limitations

This study has some limitations. First, the data used in this study were from a cohort of clinical nurses at general and university hospitals, which prevented a more diverse analysis by hospital type and organizational environment, including working conditions, annual salary, and job satisfaction. Second, numerous cases of missing data arose due to incomplete questionnaire responses. This missing data affected the ability to define the survival time of clinical nurses and the unrecorded total clinical career by determining the time of either survival or censoring. Finally, the health status variable records subjective data as it is self-evaluated, and thus can be a limitation of this study.

## 5. Conclusions

In this study, we evaluated nurses’ survival time in their career of nursing and analyzed the differences in survival time according to sleep quality and presenteeism to identify the factors that affect nurse turnover.

Irrespective of career duration, many clinical nurses showed different physical and psychological responses to their work depending on their health status. Approaches that promote a healthier lifestyle for clinical nurses are needed to decrease the turnover rate of clinical nurses and increase their survival time. These approaches include improving the pattern and quality of sleep for newly registered nurses and relieving work stress and emotional fatigue due to health issues for existing nurses who are more experienced. Guidelines should be developed to prevent turnover during this period of initial adaptation. Further studies should be conducted to investigate other factors associated with the turnover of clinical nurses based on career duration, including the nursing environment and assigned tasks.

## Figures and Tables

**Table 1:** Results of an analysis of differences in general characteristics and survival time for turnover vs. non-turnover groups.

Variable	Total (*n* = 857)	Turnover (*n* = 284)	Non-Turnover (*n* = 573)	χ^2^ or *t*	*p*	Survival (Months)		
	*n* (%) or M ± SD	*n* (%) or M ± SD	*n* (%) or M ± SD			Median Survival	95% CI	*p*
Gender								
Men	33 (3.9)	11 (3.9)	22 (3.8)	0.027	0.869	.	.	0.487
Women	824 (96.1)	273 (96.1)	551 (96.2)			133.0	133.0~141.0	
Age (years)	29.1 ± 4.5	29.0 ± 5.1	29.1 ± 4.2	−0.350	0.726			
Marital status								
Married	232 (27.1)	64 (22.5)	168 (29.3)	4.09	0.043	143.0	.	<0.001
Single	625 (72.9)	220 (77.5)	405 (70.7)			123.0	109.0~133.0	
Religion								
Yes	385 (44.9)	131 (46.1)	254 (44.3)	0.181	0.671	134.0	128.0~143.0	0.748
No	472 (55.1)	153 (53.9)	319 (55.7)			133.0	130.0~141.0	
Educational background								
<3 y	280 (32.7)	88 (31.0)	192 (33.5)	0.44	0.507	141.0	133.0~143.0	0.002
≥4 y	577 (67.3)	196 (69.0)	381 (66.5)			133.0	121.0~134.0	
Department								
Internal medicine	454 (53.0)	147 (51.8)	307 (53.6)	0.184	0.668	133.0	133.0~142.0	0.519
Surgical	403 (47.0)	137 (48.2)	266 (46.4)			133.0	115.0~143.0	
Turnover plan								
Yes	475 (55.4)	194 (68.3)	281 (49.0)	27.765	<0.001	121.0	104.0~133.0	<0.001
No	382 (44.6)	90 (31.7)	292 (51.0)			143.0	.	
Health status								
Good	517 (60.3)	151 (53.2)	366 (63.9)	8.651	0.003	142.0	134.0~143.0	0.003
Poor	340 (39.7)	133 (46.8)	207 (36.1)			130.0	115.0~133.0	
Region								
Capital area	625 (72.9)	214 (75.4)	411 (71.7)	1.087	0.297	133.0	130.0~141.0	0.427
Provinces	232 (27.1)	70 (24.6)	162 (28.3)			143.0	133.0~143.0	
Nursing career								
≤3 y; Competent nurses	38 (4.4)	17 (6.0)	21 (3.7)	19.975	<0.001	33.0	29.0~34.0	<0.001
3~6 y; Proficient nurses	300 (35.0)	125 (44.0)	175 (30.5)			64.0	61.0~67.0	
>6 y; Expert nurses	519 (60.6)	142 (50.0)	377 (65.8)			143.0	134.0~143.0	
Length of clinical career (months)	82.8 ± 32.7	76.1 ± 32.7	86.2 ± 32.2	−4.284	<0.001			

Abbreviations: CI, Confidence interval; SD, standard deviation.

**Table 2:** Results of the time-independent Cox proportional hazard regression.

Variable	HR	95% CI	*p*
Total			
Sleep_score	1.28	(1.02, 1.60)	0.03
Presenteeism score	1.14	(0.94, 1.37)	0.17
Goodness-of-fit Test			
LR test			<0.01
Wald test			<0.01
Score test			<0.01
3 years or less			
Sleep_score	9.16	(2.41, 34.73)	<0.01
Presenteeism score	1.0		
Goodness-of-fit Test			
LR test			<0.01
Wald test			<0.01
Score test			<0.01
3–6 years			
Sleep_score	1.0		
Presenteeism score	1.0		
More than 6 years			
Sleep_score	1.0		
Presenteeism score	1.31	(1.02, 1.68)	0.03
Goodness-of-fit Test			
LR test			<0.01
Wald test			<0.01
Score test			<0.01
Total subitem analysis			
Sleep_01	1.15	(0.98, 1.36)	0.09
Presenteeism_05	1.13	(1.02, 1.27)	0.02
Goodness-of-fit Test			
LR test			<0.01
Wald test			<0.01
Score test			<0.01
3 years or less subitem analysis			
Sleep_01	2.92	(1.13, 7.53)	0.03
Sleep_03	2.17	(1.35, 3.48)	<0.01
Goodness-of-fit Test			
LR test			<0.01
Wald test			<0.01
Score test			<0.01
3–6 years subitem analysis			
Presenteeism_04	1.17	(1.00, 1.37)	0.06
Goodness-of-fit Test			
LR test			0.06
Wald test			0.05
Score test			0.05
More than 6 years subitem analysis			
Presenteeism_05	1.22	(1.05, 1.41)	0.01
Goodness-of-fit Test			
LR test			<0.01
Wald test			<0.01
Score test			<0.01

Abbreviations: CI, confidence interval; HR, hazard ratio; LR, likelihood ratio. HR 1.0, *p*-value: removed from stepwise selection by partial log-likelihood. Sleep_01: Subjective sleep quality. Sleep_03: Sleep time. Presenteeism_04: I had health problems that bothered me, but I had enough energy to get the job done. Presenteeism_05: I had difficulty controlling work stress as I was concerned with a health issue.

## Data Availability

The data used is confidential, and the study participants have not consented to data sharing. Due to the sensitive nature of the personal health information and job outcomes in the questions asked in this study, the survey respondents were assured that the raw data would be kept confidential and would not be shared.

## References

[1] Lee T., Kang K.H., Ko Y.K., Cho S.-H., Kim E.-Y. (2014). Issues and challenges of Nurse Workforce Policy: A critical review and implication. J. Korean Acad. Nurs. Admin..

[2] Kim J., Bae H., Jeong S. (2017). Forecasting supply and demand for registered nurses workforce in Korea. Korean Data Anal. Soc..

[3] (2022). Ministry of Health and Welfare Survey on Health and Medical Personnel. https://kosis.kr/statHtml/statHtml.do?orgId=117&tblId=DT_117110_E005&vw_cd=MT_ZTITLE&list_id=F_007_005&seqNo=&lang_mode=ko&language=kor&obj_var_id=&itm_id=&conn_path=MT_ZTITLE.

[4] Hospital Nurses Association (2019). A Survey on Hospital Nursing Staffing 2018.

[5] Labrague L.J., los Santos J.A.A., Falguera C.C., Nwafor C.E., Galabay J.R., Rosales R.A., Firmo C.N. (2020). Predictors of nurses’ turnover intention at one and five years’ time. Int. Nurs. Rev..

[6] Yoo M.S., Jeong M.R., Kim K., Lee Y. (2019). Factors influencing differences in turnover intention according to work periods for newly graduated nurses. J. Korean Acad. Nurs. Admin..

[7] Whitehead L., Ghosh M., Walker D.K., Bloxsome D., Vafeas C., Wilkinson A. (2019). The relationship between Specialty Nurse Certification and patient, nurse and organizational outcomes: A systematic review. Int. J. Nurs. Stud..

[8] Griffiths P., Maruotti A., Recio Saucedo A., Redfern O.C., Ball J.E., Briggs J., Dall’Ora C., Schmidt P.E., Smith G.B. (2018). Nurse staffing, nursing assistants and hospital mortality: Retrospective longitudinal cohort study. Br. Med. J. Qual. Safe.

[9] Feng H., Qi X., Xia C.L., Xiao S., Fan L. (2021). Association between night shift and sleep quality and health among Chinese nurses: A cross-sectional study. J. Nurs. Manag..

[10] Kecklund G., Axelsson J. (2016). Health consequences of shift work and insufficient sleep. Br. Med. J..

[11] ten Hoeve Y., Kunnen S., Brouwer J., Roodbol P.F. (2018). The voice of nurses: Novice nurses’ first experiences in a clinical setting. A longitudinal diary study. J. Clin. Nurs..

[12] Yu M., Choi-Kwon S. (2020). Secondary data analysis on the quality of sleep and related factors of novice and experienced shift work nurses. J. Korean Acad. Nurs..

[13] Han K., Kim Y.-H., Lee H.Y., Lim S. (2020). Novice nurses’ sleep disturbance trajectories within the first 2 years of work and actual turnover: A prospective longitudinal study. Int. J. Nurs. Stud..

[14] Johns G. (2009). Presenteeism in the workplace: A review and research agenda. J. Org. Behav..

[15] Kim M., Choi H.O., Ryu E. (2014). Predictors of clinical nurses’ presenteeism. Korean J. Occupat. Health Nurs..

[16] Freeling M., Rainbow J.G., Chamberlain D. (2020). Painting a picture of nurse presenteeism: A multi-country integrative review. Int. J. Nurs. Stud..

[17] Rainbow J.G., Gilbreath B., Steege L.M. (2019). How to know if you’re really there: An evaluation of measures for presenteeism in nursing. J. Occupat. Environ. Med..

[18] Rainbow J.G., Gilbreath B., Steege L.M. (2020). Risky business: A mediated model of antecedents and consequences of presenteeism in nursing. Nurs. Res..

[19] Homrich P.H., Dantas-Filho F.F., Martins L.L., Marcon E.R. (2020). Presenteeism among health care workers: Literature review. Rev. Bras. Med. Trab..

[20] Alhamwan M., Mat N.B., Muala I.A. (2015). The impact of organizational factors on nurses turnover intention behavior at public hospitals in Jordan: How does leadership, career advancement and pay-level influence the turnover intention behavior among nurses. J. Manag. Sustain.

[21] Kim C., Lee Y. (2020). Effects of compassion competence on missed nursing care, professional quality of life and quality of life among Korean nurses. J. Nurs. Manag..

[22] Benner P. (1984). From Novice to Expert.

[23] Sohn S.I., Kim D.H., Lee M.Y., Cho Y.W. (2012). The reliability and validity of the Korean version of the Pittsburgh Sleep Quality index. Sleep Breath.

[24] Buysse D.J., Reynolds C.F., Monk T.H., Berman S.R., Kupfer D.J. (1989). The Pittsburgh Sleep Quality index: A new instrument for psychiatric practice and Research. Psychiatry. Res..

[25] Turpin R.S., Ozminkowski R.J., Sharda C.E., Collins J.J., Berger M.L., Billotti G.M., Baase C.M., Olson M.J., Nicholson S. (2004). Reliability and validity of the Stanford presenteeism scale. J. Occupat. Environ. Med..

[26] Han K., Kim Y.-H., Lee H.Y., Lim S. (2019). Pre-employment health lifestyle profiles and actual turnover among newly graduated nurses: A descriptive and prospective longitudinal study. Int. J. Nurs. Stud..

[27] Cho S.-H., Lee J.Y., Mark B.A., Yun S.-C. (2012). Turnover of new graduate nurses in their first job using survival analysis. J. Nurs. Schol..

[28] Cao T., Huang X., Wang L., Li B., Dong X., Lu H., Wan Q., Shang S. (2020). Effects of organisational justice, work engagement and nurses’ perception of care quality on turnover intention among newly licensed registered nurses: A structural equation modelling approach. J. Clin. Nurs..

[29] Ki J., Ryu J., Baek J., Huh I., Choi-Kwon S. (2020). Association between health problems and turnover intention in shift work nurses: Health problem clustering. Int. J. Environ. Res. Public Health.

[30] Membrive-Jiménez M.J., Gómez-Urquiza J.L., Suleiman-Martos N., Velando-Soriano A., Ariza T., la Fuente-Solana D., Inmaculada E. (2020). Relation between burnout and sleep problems in nurses: A systematic review with meta-analysis. Healthcare.

[31] Palermo T.A.D.C., Rotenberg L., Zeitoune R.C.G., Silva-Costa A., Souto E.P., Griep R.H. (2015). Napping during the night shift and recovery after work among hospital nurses. Rev. Lat. Am. Enfermagem.

[32] Kang J., Noh W., Lee Y. (2020). Sleep quality among shift-work nurses: A systematic review and meta-analysis. Appl. Nurs. Res..

[33] Frögéli E., Rudman A., Ljótsson B., Gustavsson P. (2018). Preventing stress-related ill health among newly registered nurses by supporting engagement in Proactive Behaviors: Development and feasibility testing of a behavior change intervention. Pilot Feas. Stud..

[34] Aiken L.H., Sermeus W., Van den Heede K., Sloane D.M., Busse R., McKee M., Bruyneel L., Rafferty A.M., Griffiths P., Moreno-Casbas M.T. (2012). Patient safety, satisfaction, and quality of hospital care: Cross sectional surveys of nurses and patients in 12 countries in Europe and the United States. Br. Med. J..

[35] Nei D., Snyder L.A., Litwiller B.J. (2015). Promoting retention of nurses: A meta-analytic examination of causes of nurse turnover. Health Care Manag. Rev..

[36] Vandenbroeck S., Van Gerven E., De Witte H., Vanhaecht K., Godderis L. (2017). Burnout in Belgian physicians and nurses. Occupat. Med..

